# Hesperidin Helps Improve the Intestinal Structure, Maintain Barrier Function, and Reduce Inflammation in Yellow-Feathered Broilers Exposed to High Temperatures

**DOI:** 10.3390/ani14172585

**Published:** 2024-09-05

**Authors:** Shaoping He, Guozhi Bian, Yuming Guo, Jiyu Guo

**Affiliations:** 1 College of Animal Science and Technology, China Agricultural University, Beijing 100193, China; heshaoping56778@163.com; 2 Animal Husbandry and Fisheries Research Center of Guangdong Haid Group Co., Ltd., Guangzhou 511400, China; biangz@haid.com.cn

**Keywords:** hesperidin, high-temperature, heat stress, broilers, intestinal morphology, barrier integrity

## Abstract

**Simple Summary:**

This study aimed to elucidate the protective effects of hesperidin on intestinal damage induced by high-temperature heat stress in yellow-feathered broilers. The findings indicate that dietary supplementation with hesperidin significantly attenuated serum corticosterone and adrenocorticotropic hormone levels elevated by heat stress. Specifically, supplementation with 300 mg/kg and 450 mg/kg of hesperidin was associated with enhanced intestinal morphology, reduced endotoxin and D-lactic acid levels, and the upregulated expression of tight junction proteins in the small intestine. Hesperidin supplementation was observed to decrease pro-inflammatory cytokine levels and *NF-κB* mRNA expression. These results indicate that hesperidin may serve as a viable intervention to mitigate intestinal damage induced by heat stress in broilers, thereby providing novel insights for enhancing broiler health and welfare.

**Abstract:**

To investigate the possible protective effect of hesperidin on intestinal damage caused by high-temperature heat stress in yellow-feathered broilers, 960 broilers aged 21 days were randomly divided into four groups: HT, HT300, HT450, and HT600, with each group receiving different amounts of hesperidin supplementation (0, 300, 450, and 600 mg/kg). The dietary supplementation of hesperidin could mitigate the elevation of corticosterone (CORT) and adrenocorticotropic hormone (ATCH) levels in serum from yellow-feathered broilers induced by heat stress. The supplementation of 300 mg/kg and 450 mg/kg of hesperidin reduced crypt depth and increased the V/C ratio in the small intestine compared to the HT group. The dietary supplementation of hesperidin decreased endotoxin and D-lactic acid levels in the blood, and dietary supplementation of 300 mg/kg of hesperidin increased the expression of *claudin-1* and *ZO-1* mRNA in the jejunum compared with the HT group. Furthermore, the dietary supplementation of 300 mg/kg of hesperidin decreased serum IL-1β and IL-6 levels. In comparison, supplementation with 300 mg/kg and 450 mg/kg of hesperidin decreased serum TNF-α levels in yellow-feathered broilers compared to the HT group. Moreover, the dietary supplementation of hesperidin decreased *NF-κB* mRNA levels. Overall, these data suggest that dietary supplementation with hesperidin potentially improves intestinal injury caused by heat stress in yellow-feathered broilers.

## 1. Introduction

Heat stress refers to the non-specific physiological response exhibited by livestock and poultry when exposed to elevated ambient temperatures that are detrimental to their normal physiological functions. This condition significantly impairs the health and productivity of these animals, leading to substantial economic losses within the breeding industry [1,2]. The annual increase in global environmental temperatures, attributable to the intensification of the greenhouse effect, has progressively exacerbated the heat stress challenges faced by numerous intensive and large-scale poultry operations [3,4]. The small body size, presence of feathers, and lack of sweat glands in poultry require them to rely on increased skin permeability and respiratory evaporation for heat dissipation [5,6]. Additionally, poultry have a high metabolic rate, leading to the buildup of excess heat when their heat generation exceeds their dissipation capacity. These inefficiencies in heat dissipation render poultry more susceptible to heat stress, hindering the growth and development of various tissues and organs in these avian species [7].

The intestinal organ in poultry is highly vulnerable to heat stress, affecting feed efficiency and conversion rates, which are essential for achieving optimal growth and economic sustainability [3]. The intestine serves as the principal site for nutrient processing and absorption and is crucial in mitigating vulnerability to heat stress and supporting normal metabolic processes within the organ [5,6]. The dysfunction of the poultry intestinal barrier induced by heat stress will disrupt intestinal microbiota balance and oxidative stress within the intestinal mucosa [5], leading to the excessive production of cytokines and intestinal inflammation [8]. Under heat stress conditions, poultry exhibit increased body surface blood flow and reduced blood flow to internal organs to facilitate heat dissipation [9]. However, this physiological response consequently diminishes the blood volume directed towards the digestive tract, reducing the availability of essential nutrients and energy necessary for maintaining cellular vitality and function [9]. In addition, ischemic injury to the intestine induced by heat stress would impair intestinal barrier function, which leads to an increase in the passage of diamine oxidase and lipopolysaccharide from the damaged site into the bloodstream, eliciting an inflammatory reaction and impacting intestinal health [10]. The gut, essential for processing and absorbing nutrients and acting as a protective barrier, is susceptible to stress caused by high temperatures. The presence of heat stress reduces oxygen and nutrient availability in intestinal tissues, causing morphological damage, the disruption of the mucosa, decreased villus height, and a lower villus-to-crypt ratio, ultimately impacting intestinal health [5].

Hesperidin, a dihydroflavone glycoside compound obtained from hesperetin and the disaccharide rutinose, is a natural phenolic compound and a significant active flavonoid present in dried tangerine peel (Chenpi) [11]. Chenpi, derived from the fully dried peel of citrus fruits belonging to the Rutaceae family, is readily available, economically feasible, and abundant [11]. Previous studies have indicated that hesperidin displays antioxidant properties and enhances the metabolism and the assimilation of nutrients [12]. In addition, dietary supplementation with hesperidin has been reported to enhance both growth performance and the quality of the meat produced in chicken [13,14]. Furthermore, adding hesperidin could improve growth performance and relieve the impact of heat stress on broilers by decreasing the expression of creatine kinase, lactate dehydrogenase, and heat shock protein 70 in elevated temperature environments [15]. This research aims to explore the possible benefits of hesperidin in reducing intestinal barrier injury in yellow-feathered broilers exposed to high temperatures.

## 2. Materials and Methods

### 2.1. Materials and Reagents

Hesperidin (≥98%) was provided by Shanghai Yuanye Bio-Technology Co., Ltd. (Shanghai, China).

### 2.2. Animal Rearing and Experiments

The study protocol was approved by the Ethics Committee of China Agricultural 93 University (approval ID: HD#20220014). A total of 960 healthy yellow-feathered broilers, aged 21 days, underwent individual weight measurements before being randomly allocated to four distinct treatment groups. All the yellow-feathered broilers were purchased from a commercial hatchery (Elite hatchery of yellow-feathered broilers, Zhuzhou, China). Each group consisted of six replicates, with 40 chickens per replicate. Heat stress was regulated by seasonal high temperature, maintaining a room temperature of 37 ± 2 °C for 6 h daily (from 11:00 to 17:00 in August), and the remaining hours were set at 24 ± 2 °C based on findings from prior research; the heat stress temperature was maintained by electric heater and air conditioner [16,17]. The group with high temperatures was given a basic diet (HT group). In contrast, the other groups were administered the standard diet augmented with varying concentrations of hesperidin (300, 450, or 600 mg/kg), designated as the HT300, HT450, and HT600 groups. The hesperidin dose was selected based on the results from the preliminary experiment. The study spanned 30 days, commencing on the 21st day of age. As shown in Table 1, the basal diet for yellow-feathered broilers was formulated following the nutritional recommendations provided by the NRC (1994).

The chickens were kept in enclosures that were 320 × 240 cm in size, with free access to food and water throughout the experiment. The broilers were monitored thrice daily to assess their well-being, feed consumption, and overall health. After the daily high-temperature treatment ended, ventilation was employed to lower the temperature. The relative humidity in the housing environment was controlled between 60% and 70%.

### 2.3. Sample Collection

The following samples were collected on day 51, the end of this study. A 10 mL blood sample was collected randomly from one chicken per replicate through the brachial vein. The serum was collected after centrifugation at 1000× *g* for 15 min at 4 °C, then aliquoted and stored at −20 °C. Intestinal samples were obtained by selecting one chicken from each replicate and anesthetizing them using ether inhalation. A 2-centimeter portion from the center of the jejunum was obtained and rinsed with PBS. About 2 grams of mucosa from the jejunum was scraped off with clean glass slides, placed in sterile aluminum foil, and quickly frozen in liquid nitrogen at −80 °C for ribonucleic acid (RNA) extraction. Furthermore, sections measuring 1 cm in length from the center of both the jejunum and ileum were excised and preserved in 10% neutral formalin at 4 °C for later examination of intestinal morphology.

### 2.4. Intestinal Permeability Test

The serum levels of endotoxin, diamine oxidase (DAO), and D-lactate were measured to assess gut permeability. According to the provided guidelines, endotoxin, DAO, and D-lactic acid levels were measured using chicken ELISA kits (MLBIO Co., Ltd., Shanghai, China). Thermo Fisher Scientific, Waltham, MA, USA was employed for the analytical determinations.

### 2.5. Serum Cytokine Levels

Serum concentrations of IL-1β, IL-4, IL-6, TNF-α, and IFN-γ were measured using chicken ELISA kits (Bogoo Bio in Shanghai, China) following the manufacturer’s instructions and employing the ELISA method from Thermo Fisher Scientific in Waltham, MA, USA.

### 2.6. Intestinal Morphology Analysis

To evaluate changes in the structure of the jejunum and ileum of broilers, samples of the intestines were processed using standard histological techniques, including dehydration, embedding in paraffin, cutting into sections, and staining with hematoxylin and eosin (H&E) based on previously studies [16,17]. One slice was extracted for analysis in each repetition. Five representative fields were chosen from each stained section for analysis. The selection of specific fields was conducted to maintain the integrity and proper alignment of the villi. Within each designated field, three villi and their corresponding crypts were assessed. The crypt’s depth was measured by determining the vertical distance between the crypt’s base and the villus-crypt junction.

### 2.7. Real-Time PCR

Real-time PCR was utilized to detect mRNA expression levels according to the procedures outlined in a prior investigation [18]. The jejunum extracted total RNA with the Steadypure Universal RNA Extraction Kit and converted it into cDNA with the Evo M-MLV RT Premix Ki. PCR reactions were carried out in a 10 μL total volume, with 10 μL of 2× SybrGreen qPCR Master Mix (SYBR^®^ Green Premix Pro Taq HS qPCR Kit, Accurate Biotechnology Co., Ltd., Changsha, Hunan, China), 0.2 μL of each forward and reverse primers (10 μmol/L), 1 μL of cDNA template, and 3.6 μL of sterile water. Ct values were examined to evaluate the consistency of the β-actin genes. Analysis of data was performed utilizing the 2−ΔΔCT technique. The primers utilized in this study are detailed in Table S1.

### 2.8. Statistical Analysis

All data were analyzed using SPSS 21.0 software (SPSS, Inc., Chicago, IL, USA), with each replicate mean serving as the unit of analysis. All data was subjected to normality and variance homogeneity tests. The impact of varying concentrations of hesperidin on broilers in high-temperature environments was assessed using ANOVA and Duncan’s multiple range test. Mean values with standard error (SE) are reported, with significance determined at *p* < 0.05.

## 3. Results

### 3.1. Impact of Hesperidin on the Growth Performance of Yellow-Feathered Broilers in High Temperatures

The growth performance of yellow-feathered broilers under heat stress conditions is shown in Table 2. The results indicated that dietary supplementation with hesperidin did not have a statistically significant impact on the average daily feed intake (ADFI), average daily gain (ADG), mortality, or ratio of feed to gain (F/G) in broilers under heat stress conditions.

Every average corresponds to a group of six replicates (*n* = 6).

### 3.2. Effect of Hesperidin on Serum Hormone Concentration of Yellow-Feathered Broilers under High Temperature

**Figure 1:**
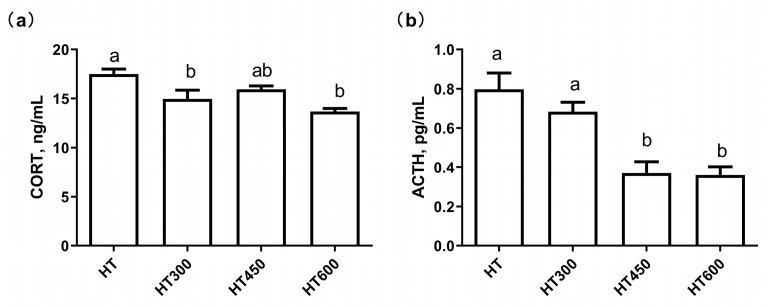
Effect of hesperidin on serum hormone concentration of yellow-feathered broilers under high temperatures. (**a**) CORT; (**b**) ACTH. HT, high-temperature treatment (37 ± 2 °C). HT300, HT + 300 mg/kg of hesperidin. CORT, corticosterone; ACTH, adrenocorticotropic hormone. Values are expressed as mean and SEM, and bars labeled with different letters significantly differ (*p* < 0.05).

Serum levels of corticosterone (CORT) and adrenocorticotropic hormone (ACTH), which serve as biomarkers for assessing heat stress, significantly increased in poultry subjected to heat stress. Figure 1 illustrates the influence of hesperidin on hormone levels in yellow-feathered broilers experiencing heat stress. Our data showed that the addition of 300 mg/kg and 600 mg/kg of hesperidin to the diet led to a significant decrease in serum CORT levels in yellow-feathered broiler chickens when compared to the HT group (*p* < 0.05) (Figure 1). Additionally, the supplementation of 450 mg/kg and 600 mg/kg of hesperidin to the diet significantly decreased serum ATCH levels in yellow-feathered broiler chickens (*p* < 0.05) compared with the HT group (Figure 1).

### 3.3. Impact of Hesperidin on the Structure of the Jejunum and Ileum in Yellow-Feathered Broilers Exposed to High Temperatures

To further evaluate the effects of hesperidin on the intestinal structure of yellow-feathered broiler chickens under heat stress, the indices of morphology of the jejunum and ileum were assessed and were outlined in Table 3. The data showed that adding 450 mg/kg of hesperidin to the diet led to a significant decrease in crypt depth in the jejunum and a significant increase in the villus-to-crypt ratio compared to the HT group (*p* < 0.05). Moreover, supplementing the diet with 450 mg/kg and 600 mg/kg of hesperidin resulted in a significant reduction in crypt depth in the ileum and a marked enhancement in the villus-to-crypt ratio in the ileum compared with the HT group (*p* < 0.05).

### 3.4. Impact of Hesperidin on Gut Permeability in Yellow-Feathered Broiler Chickens Exposed to High Temperatures

**Figure 2:**
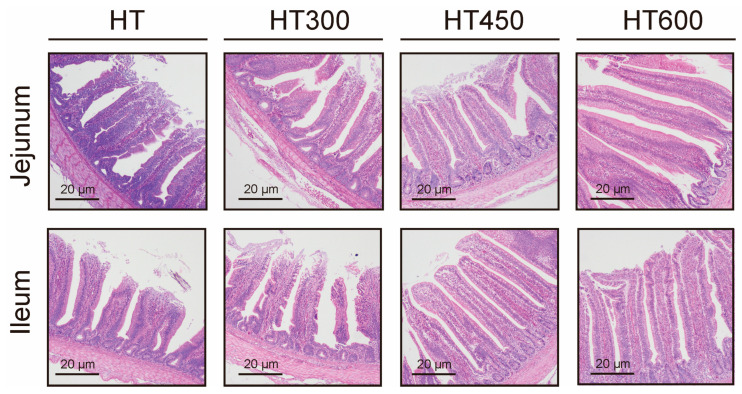
Effect of hesperidin on the intestine morphology of yellow-feathered broilers under high temperatures.

**Figure 3:**
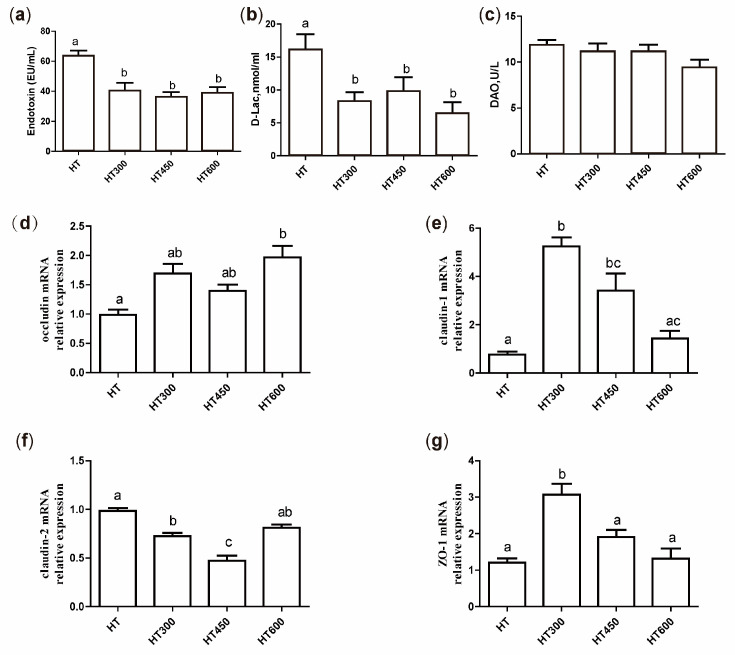
Effect of hesperidin on the intestinal permeability of yellow-feathered broilers under high temperatures. Effect of hesperidin on serum levels of endotoxin (**a**), D-Lac (**b**), and DAO (**c**) of yellow-feathered broilers under high temperatures. The mRNA expression of *occludin*, *claudin-1*, *claudin-2*, and *ZO-1* is shown in (**d**–**g**). Mean and SEM values are presented, with bars denoted by distinct letters indicating significant differences (*p* < 0.05). HT, high-temperature processing at 37 ± 2 °C. HT300, HT + 300 mg/kg of hesperidin.

Endotoxin, D-Lac, and DAO are biomarkers that indicate intestinal permeability and intestinal damage. As shown in Figure 2, the dietary supplementation of hesperidin led to a notable decrease in serum endotoxin and D-lactate levels in yellow-feathered broiler chickens (*p* < 0.05) (Figure 3a,b) but had no significant effect on DAO (Figure 3c). In addition, real-time PCR showed that the *occludin* mRNA expression in the jejunum increased significantly when 600 mg/kg of hesperidin was added to the diet (*p* < 0.05) (Figure 3d). Furthermore, the supplementation of 300 mg/kg and 450 mg/kg of hesperidin in the diet remarkably elevated jejunal *claudin-1* mRNA expression (*p* < 0.05) (Figure 3e) and reduced jejunal *claudin-2* mRNA expression (*p* < 0.05) (Figure 3f). Adding 300 mg/kg of hesperidin to the diet also significantly increased jejunal *ZO-1* mRNA expression (Figure 3g).

### 3.5. Impact of Hesperidin on the Intestinal Mucosa of Yellow-Feathered Broiler Chickens during Periods of Heat Stress

**Figure 4:**
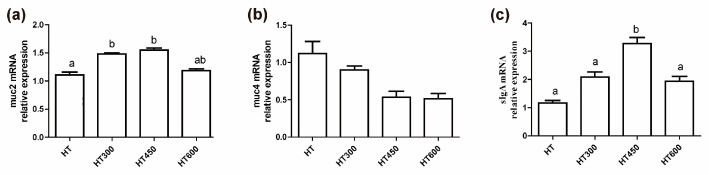
Impact of hesperidin on intestinal mucosa in yellow-feathered broiler chickens experiencing heat stress. The levels of mucins and *sIgA* gene mRNA are shown in (**a**–**c**). Mean and SEM values are presented, with bars denoted by distinct letters indicating significant differences (*p* < 0.05). HT, high-temperature processing at 37 ± 2 °C. HT300, HT + 300 mg/kg of hesperidin.

As shown in Figure 4, the supplementation of 300 mg/kg and 450 mg/kg of hesperidin resulted in a notable rise in jejunal *muc2* mRNA expression (*p* < 0.05) (Figure 4a). However, there was no significant effect on *muc4* mRNA expression (Figure 4b). Meanwhile, adding 450 mg/kg of hesperidin to the diet led to a notable rise in jejunal *sIgA* mRNA expression, with statistical significance (*p* < 0.05) (Figure 4c).

### 3.6. Impact of Hesperidin on Cytokine Levels in the Serum of Yellow-Feathered Broilers Exposed to High Temperatures

**Figure 5:**
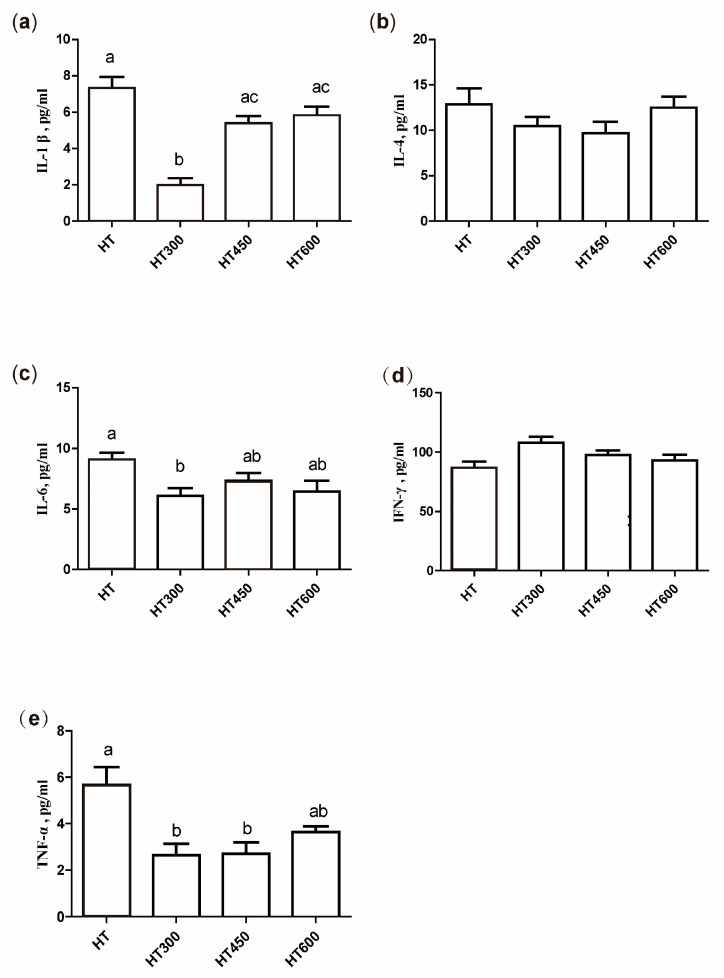
Impact of hesperidin on cytokine levels in the serum of yellow-feathered broilers exposed to high temperatures. IL-1β (**a**), IL-4 (**b**), IL-6 (**c**), IFN-γ (**d**), TNF-α (**e**). Mean and SEM values are presented, with bars denoted by distinct letters indicating significant differences (*p* < 0.05). HT, high-temperature processing at 37 ± 2 °C. HT300, HT + 300 mg/kg of hesperidin.

The impact of hesperidin on serum inflammatory cytokine levels in heat stress conditions is depicted in Figure 5. The addition of 300 mg/kg of hesperidin led to a notable reduction in serum IL-1β and IL-6 levels compared to the HT group (*p* < 0.05) (Figure 5a,c). Furthermore, the addition of both 300 mg/kg and 450 mg/kg of hesperidin led to a significant reduction in serum TNF-α levels (*p* < 0.05) (Figure 5e). However, the dietary supplementation of hesperidin exhibited no significant influence on serum IL-4 and IFN-γ levels in yellow-feathered broilers exposed to high temperatures.

### 3.7. Impact of Hesperidin on the HSP70, HSP90, and NF-κB mRNA Levels in the Jejunum of Yellow-Feathered Broilers Exposed to High Temperatures

**Figure 6:**
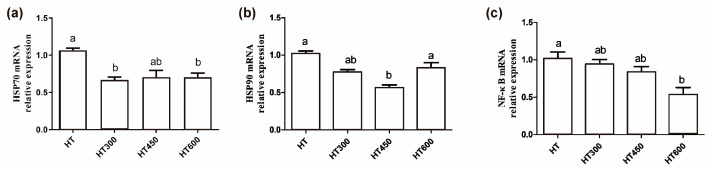
Impact of hesperidin on mRNA expression levels in the jejunum of yellow-feathered broilers exposed to high temperatures. The mRNA levels of *HSP70*, *HSP90*, and *NF-κB* genes are depicted in panels (**a**–**c**). Mean and SEM values are presented, with bars denoted by distinct letters indicating significant differences (*p* < 0.05). HT, high-temperature processing at 37 ± 2 °C. HT300, HT + 300 mg/kg of hesperidin.

As shown in Figure 6, the supplementation of 300 mg/kg and 600 mg/kg of hesperidin in the diet led to a notable reduction in jejunal *HSP70* mRNA expression compared to the HT group (*p* < 0.05) (Figure 6a). Moreover, the inclusion of 450 mg/kg of hesperidin resulted in a notable decrease in jejunal *HSP90* mRNA levels (*p* < 0.05) (Figure 6b). Supplementation with 600 mg/kg of hesperidin led to a notable reduction in jejunal *NFκB* mRNA expression (*p* < 0.05) compared to the control group (Figure 6c).

## 4. Discussion

### 4.1. The Influence of Hesperidin on the Growth Performance of Yellow-Feathered Broilers in High Temperatures

Heat stress markedly reduced ADFI and ADG while significantly elevating the F:G. A recent study on yellow-feathered broilers subjected to heat stress reported a substantial decrease in body weight gain (−31.85%), feed intake (−15.8%), and the weight gain to feed consumption ratio (−18.8%) during the period of 22 to 42 days [17]. This phenomenon can be attributed to the increased energy expenditure by broilers for maintenance and acclimatization to stressful conditions. Consequently, reduced metabolic rate reduces nutrient deposition and suboptimal performance in heat-stressed broilers. Hesperidin, a flavonoid derived from citrus fruits, has been shown to possess growth-promoting and antioxidant characteristics [11]. A study conducted by Kamboh et al. (2013) indicated that the supplementation of 20 mg/kg of hesperidin in the diet during periods of seasonal heat stress resulted in enhanced broiler growth performance and mitigation of heat stress [15]. Moreover, when broilers were subjected to LPS-induced inflammation, the supplementation of 20 mg/kg of hesperidin would increase overall antioxidant capacity (TAOC) and superoxide dismutase (SOD) function while also decreasing malondialdehyde production. This treatment also facilitated the development of intestinal villi and exhibited reparative effects on intestinal damage. The inconsistent result may be related to the period time of hesperidin nutritional intervention. In previous study, hesperidin was provided over entire lifespan of broilers, from day 1 to day 42, by which it may have more accurately impact on the growth performance.

### 4.2. The Influence of Hesperidin on the Serum Hormone Concentration of Yellow-Feathered Broilers under High-Temperature

When chickens are exposed to intense heat, it causes the neuroendocrine system to become active, particularly the hypothalamic–pituitary–adrenal axis. Corticotropin-releasing hormone (CRH) is released from the hypothalamus, and adrenocorticotropic hormone (ACTH) is released from the pituitary gland in response to heat stress. As a result, ACTH promotes the transformation of cholesterol into the CORT, which then triggers the synthesis and secretion of steroids, causing an increase in plasma CORT levels [19]. The presence of hesperidin reduced serum ACTH levels, indicating that hesperidin could help mitigate the endocrine and metabolic disturbances induced by heat stress. Previous studies have demonstrated that flavonoid supplements effectively reduce heat stress in poultry [20,21]. Nevertheless, some studies have indicated that including 20 mg/kg of hesperidin in the diet enhanced the growth of broilers. In contrast, the dosages administered in this experiment were elevated, varying between 300 and 600 mg/kg. The elevated levels of hesperidin administered in this study may have heightened the basal metabolism of broilers, potentially elucidating the absence of notable growth-promoting outcomes [11].

### 4.3. Impact of Hesperidin on Intestinal Injury in Yellow-Feathered Broiler Chickens Exposed to High Temperatures

The intestinal tract, serving as the primary organ for nutrient absorption, plays a crucial role in the growth of poultry. This study investigated the protective effects of hesperidin on intestinal damage in yellow-feathered broilers subjected to heat stress, utilizing multiple indices for assessment. Endotoxin and D-lactate are commonly acknowledged as markers of heat stress-induced intestinal barrier impairment [22]. The elevation of these markers in serum can be attributed to physiological and environmental stressors. In the gut, endotoxin, an essential component of the outer layer of Gram-negative bacteria, and D-lactate, a product of bacterial fermentation in the intestines, are commonly present [23,24]. Damage to the intestinal barrier caused by heat stress can lead to the movement of LPS and D-lactate into the bloodstream [5,19]. DAO, an intracellular enzyme in intestinal villi, is released into circulation following damage to villus cells [25]. Hesperidin reduced crypt depth in the jejunum and ileum and increased the villus-to-crypt ratio, which is essential for intestinal digestion and absorption. Heat stress has significantly increased the expression of heat shock proteins, particularly in the jejunum with high translation activity [5], which may serve as a general protective mechanism for cells’ response to heat stress. Heat shock proteins, acting as chaperones, are crucial in maintaining mitochondrial membrane integrity and regulating epithelial cell apoptosis [26]. The findings suggest that hesperidin has a beneficial effect on improving gut health in chickens experiencing heat stress.

The intestinal barrier comprises four protective layers: the microbial layer, the mucosal layer, the epithelial cell layer, and the immune barrier [27]. The mucosal layer acts as the main protective barrier of the natural host, mainly by producing mucin-2 and other active substances through intestinal goblet cells. Furthermore, secretory IgA (sIgA) can directly interact with the microbial population on the outer mucosal layer [28]. In a prior investigation, it was reported that being subjected to high temperatures led to a notable drop in mucin-2 and sIgA mRNA levels in the jejunum of broilers but an increase in mucin-4 mRNA expression [17]. Exposure to high temperatures has been demonstrated to cause a decline in goblet cells and lymphocytes in the jejunum, decreasing the production of mucin-2 and sIgA [29]. Our data showed that adding hesperidin can boost mucin-2 and sIgA mRNA levels and reduce mucin-4 mRNA levels in the jejunum of heat-exposed broilers, indicating that hesperidin effectively alleviates the harm to the mucosal layer from heat stress. Maintaining the function of the epithelial cell barrier in the intestines depends on tight junction proteins such as Claudin, ZO-1, and Occludin, which are crucial for the integrity of the mechanical barrier [30]. The disruption of intestinal tight junctions leads to an increase in intestinal permeability [30]. Claudin-1 and ZO-1 play a crucial role as tight junction proteins in the intestinal epithelium, aiding cell-to-cell communication and creating a selective barrier that controls paracellular transport [31]. Conversely, Claudin-2 acts as a pore-forming protein, enhancing extracellular permeability [31]. Studies have shown that exposure to high temperatures reduces the mRNA levels of Claudin-1, Occludin, ZO-1, and specific junctional adhesion molecules [32]. Our study showed that adding hesperidin to the diet resulted in a notable increase in the levels of Claudin-1, ZO-1, and Occludin mRNA in the jejunum of heat-exposed broilers while also reducing the levels of Claudin-2 mRNA. These results indicate that hesperidin can successfully reduce the harmful impacts of heat stress on the integrity of the intestinal barrier.

### 4.4. The Mechanism by Which Hesperidin Facilitates the Repair of Intestinal Injury Induced by Heat Stress

Cytokines are considered to play a role in thermoregulation and the development of heat stress. The changes in cytokine concentrations have been shown to be involved in a systemic inflammatory response, leading to a syndrome of multi-organ dysfunction [17]. Heat stress-induced inflammation was observed to modulate cytokine levels in the plasma, resulting in a shift from a Th1-dominant to a Th2-dominant immune response [33]. During this change, there are increased amounts of IL-1β, IL-4, IL-6, and TNF-α, along with reduced levels of IFN-γ. In the current research, we found that adding hesperidin led to lower levels of IL-1β, IL-6, and TNF-α, with no notable effect on IL-4 and IFN-γ levels. Due to the recognized suppressive impacts of hesperidin, a type of flavonoid, on the NF-κB pathway in multiple research studies, we further examined the NF-κB pathway expression. Prior research has indicated that heat stress leads to increased NF-κB expression levels and influences the expression of associated genes [17]. When cells are exposed to different stressors like cytokines, pathogens, or toxins, IκB becomes phosphorylated, leading to the release of NF-κb [34], and then moves to the nucleus and acts as a transcription factor, regulating the transcription of specific genes, such as pro-inflammatory cytokines, to adjust the cellular physiological reaction [35]. Our data demonstrate that hesperidin can suppress the mRNA expression of NF-κB in the jejunum during periods of heat stress. The inhibition is thought to contribute to the reduction of pro-inflammatory cytokines, suggesting that hesperidin could help reduce the inflammatory response caused by heat stress and protect the intestinal health of poultry.

## 5. Conclusions

In conclusion, the results of the present study indicated diet supplementation with hesperidin was a high-efficiency way of alleviating the heat stress impacts. Hesperidin could inhibit the higher HSP expression, antagonize the NF-kB and the accompanied reducing inflammatory cytokines, and initiate the expression of TJ mRNA, alleviating heat stress-induced impairment of the intestine and leading to the better performance of broilers. The mechanisms of hesperidin-regulated expression of HSPs and TJ and cytokines expressed under heat stress need further investigation.

## Figures and Tables

**Table 1:** The composition and nutrient content of the basic diets on a dry basis.

Item	Amount
Ingredient (%)	
Corn	59.50
Soybean meal	24.70
Sunflower meal	3.00
Soybean oil	6.20
Corn gluten meal	2.00
Dicalcium phosphate	1.10
Limestone	1.40
Methionine	0.2
Salt	0.30
Vitamin-mineral-premix ^a^	1.6
Total	100.00
Nutrient level ^b^	
ME/(MJ/kg)	13.00
CP (%)	19.00
Ca (%)	0.85
Available P (%)	0.40
Lysine (%)	1.1
Methionine (%)	0.50

^a^ The vitamin and mineral mixture contains the following amounts per kilogram of diet: 12,000 IU of vitamin A, 3000 IU of vitamin D3, 25 IU of vitamin E, 2.2 mg of vitamin K3, 3 mg of vitamin B1, 8 mg of vitamin B2, 4 mg of vitamin B6, 0.02 mg of vitamin B12, 45 mg of nicotinic acid, 12.5 mg of pantothenic acid, 0.1 mg of biotin, 1 mg of folic acid, 8 mg of copper, 80 mg of iron, 60 mg of zinc, 100 mg of manganese, 0.15 mg of selenium, and 0.35 mg of iodine; ^b^ Nutrient level: Lysine and Methionine were calculated values, whereas others were analyzed values.

**Table 2:** Impact of hesperidin on the growth performance of yellow-feathered broilers in high temperatures.

Item	HT	HT300	HT450	HT600	SEM	*p*-Value
Initial weight	390.33	391.83	393.00	390.66	12.81	0.879
ADG, g	41.93	42.18	41.91	41.22	2.48	0.73
ADFI, g	98.73	96.3	96.73	96.25	2.48	0.37
F/G	2.33	2.28	2.32	2.33	0.11	0.70
Mortality, %	98.75	99.58	99.16	99.16	0.30	0.14

HT, high-temperature processing at 37 ± 2 °C. HT300, HT + 300 mg/kg of hesperidin. ADFI: average daily feed intake, ADG: average daily gain, and F/G: ratio of feed to gain.

**Table 3:** Impact of hesperidin on the structure of the jejunum and ileum in yellow-feathered broilers exposed to high temperatures.

Items	HT	HT300	HT450	HT600	SEM	*p*-Value
Jejunum						
villus height (um)	75.31	92.94	104.84	88.95	56.73	0.34
Crypt depth (um)	119.30 ^a^	102.10 ^ab^	88.60 ^b^	108.60 ^ab^	3.48	0.008
V/C	6.42 ^b^	9.51 ^ab^	12.0 ^a^	8.17 ^ab^	0.73	0.04
Ileum						
villus height (um)	98.75	123.65	108.81	122.64	45.68	0.16
Crypt depth (um)	16.24 ^a^	10.46 ^b^	10.27 ^b^	13.07 ^ab^	8.19	0.02
V/C	6.63 ^b^	12.16 ^a^	10.69 ^a^	9.62 ^ab^	0.63	0.006

HT, high-temperature processing at 37 ± 2 °C. HT300, HT + 300 mg/kg of hesperidin. Mean and SEM values are presented, with bars denoted by distinct letters indicating significant differences (*p* < 0.05).

## Data Availability

The original contributions presented in the study are included in the article/Supplementary Material, further inquiries can be directed to the corresponding author/s.

## Supplementary Materials

The following supporting information can be downloaded at: https://www.mdpi.com/article/10.3390/ani14172585/s1, Table S1: Primer sequence used for the detection of mRNA levels.
