# Supplementary material for: Hesperidin Helps Improve the Intestinal Structure, Maintain Barrier Function, and Reduce Inflammation in Yellow-Feathered Broilers Exposed to High Temperatures
Source: Animals (Basel). 2024 Sep 5;14(17):2585. doi: 10.3390/ani14172585 (PMC11394609; doi:10.3390/ani14172585)
Supplement: Supplementary file 1 [file animals-14-02585-s001.zip › animals-3154123-supplementary.pdf]

Table S1.Primer sequence used for the detection of mRNA levels.

Gene	Primer sequence (5′-3′)	Product length (bp)
HSP70	F: CTGGCAATAAGCGAGCAGTGAGG<br>R: AATGCTGGCTTGCGTGGAAGAG	86
HSP90	F: GTTGCGGAGAAGGTGACAGTGATC<br>R: CTCCTTGATTCGCCGTTCTTCCAG	186
NF-κB	F: CCCCCGGCGCATTGCAGT<br>R: CCCCCACCCCATCGTCGAACT	176
Mucin-2	F: CTGCTGTGCTCCACCATTAAGTCC<br>R: GCTTGACACGCTCGGAGTATAACG	127
Mucin-4	F: ACACAGGCATCAACAGGCTCAATC<br>R: GATGTGGTGCTGGTAGTGCTGAC	112
sIgA	F: GTGAGGAGCGTGTGCTGGTT<br>R: GCTCTGCTGGGCCTGGTATG	260
Occludin	F: TACGGCAGCACCTACCTCAA<br>R: AGGCAGAGCAGGATGACGAT	107
Claudin-1	F: GCCACGTCATGGTATGGCAA<br>R: CCAGCCAATGAAGAGGGCTG	102
Claudin-2	F: TGGTGCTGCGAGATTTCCAC<br>R: CAGGGAGGAGATGATGCCCA	92
ZO-1	F: GCCAGCCATCATTCTGACTCCAC<br>R: GTACTGAAGGAGCAGGAGGAGGAG	172
β-actin	F: ATGATGATATTGCTGCGCTCGT<br>R: CCCATACCAACCATCACACCCT	139
